# Assessment of Cow’s milk-related symptom scores in early identification of cow’s milk protein allergy in Chinese infants

**DOI:** 10.1186/s12887-019-1563-y

**Published:** 2019-06-10

**Authors:** Yongmei Zeng, Jiyong Zhang, Guoqing Dong, Peihui Liu, Fei Xiao, Weiyan Li, Liting Wang, Qianzhen Wu

**Affiliations:** 1 The First Affiliated Hospital, Jinan University, Guangzhou, 510630 Guangdong China; 2 Department of Pediatrics, Shenzhen Maternity and Child Healthcare Hospital, 2004# Hongli Road, Futian District, Shenzhen, 518000 Guangdong China

**Keywords:** CoMiSS allergy food allergy food hypersensitivity Oral food challenge infant

## Abstract

**Background:**

The diagnosis of cow’s milk protein allergy(CMPA) may be easily misdiagnosed due to its lack of specific symptoms. Thus, experts have proposed the use of Cow’s milk-related symptom scores (CoMiSS) to predict CMPA. There has been no relevant report on the clinical application value of CoMiSS in Chinese children. This study aimed to evaluate the effect of CoMiSS in early identification of CMPA in Chinese infants.

**Methods:**

We calculated CoMiSS for 38 infants with suspected CMPA diagnosed in the pediatric gastroenterologic clinic in our hospital. After 4 weeks of dietary elimination and symptomatic improvement, these infants returned to our hospital to undergo oral food challenge (OFC). The ROC curve was used to determine the sensitivity and specificity of CoMiSS and evaluate the effect of CoMiSS in early identification of CMPA in Chinese infants. We didn’t determine the CoMiSS of presumed healthy infants as control group.

**Results:**

Of 38 infants who underwent OFC testing, the average CoMiSS of infants with positive OFC testing was 7.4 ± 2.3, while the average CoMiSS of infants with negative OFC testing was 4.1 ± 1.6, and there was a significant difference between two groups(*F =* 2.13, *P<*0.05). The area under the ROC curve (AUC) of CoMiSS was 0.89, and the best diagnostic cut-off point was 5.5. The sensitivity of CoMiSS was 87.5%, while the specificity of CoMiSS was 78.6%.

**Conclusion:**

CoMiSS is a simple and operable method to screen for CMPA, though there may be a risk of under-diagnosis when CoMiSS≥12 is used as the criterion for early identification of CMPA in Chinese infants. More multi-center studies are needed to evaluate whether the factors such as bloody stool should be included in CoMiSS or CoMiSS≥6 can be used as the criterion for early identification of CMPA in Chinese infants.

## Background

Cow’s milk protein allergy (CMPA) has become a public health problem worldwide and affects the health of 8% of children [1]. According to reports [2], the prevalence of CMPA in infants under one year old is between 2.0 and 7.5%, and there are no accurate epidemiological data in China. CMPA in infants is mainly manifested as symptoms in the skin, digestive tract and respiratory tract. The misdiagnosis of CMPA easily occurs due to its lack of specific symptoms, and the diagnostic rate of CMPA based on clinical experiences is higher than the actually confirmed diagnostic rate of CMPA. This can lead to unnecessary dietary avoidance in affected infants, which affects the growth and development and may increase the stress and economic burden for the families.

In September 2014, 18 experts from 14 hospitals around the world reached consensus on symptom score of CMPA and proposed the use of Cow’s milk-related symptom scores (CoMiSS) to predict CMPA. CoMiSS ≥12 points was proposed to diagnose CMPA [3]. There is no relevant report on the clinical application value of CoMiSS in Chinese children. Thus, we determined CoMiSS and carried out oral food challenge (OFC) testing to confirm or exclude CMPA and evaluated the sensitivity and specificity of CoMiSS in early identification of CMPA in Chinese infants.

## Methods

### Population

We carried out a prospective study which was conducted in pediatric gastrointestinal clinic in our hospital from June 2016 to May 2017. We enclosed all infants 1–12 month of age who were consulted in our pediatric gastrointestinal clinic. Infants with suspected CMPA were assessed with CoMiSS and underwent OFC testing. Suspected CMPA was defined as documented dietary exposure of infants and their mothers to cow’s milk protein plus at least one of the following conditions: (1) anaphylactic reaction (2) gastrointestinal, respiratory, or dermatologic manifestations, excluding acute infection diseases. All participating physicians received standard training for the study (determination of CoMiSS, oral challenge test protocol, and CMPA diagnosis and treatment guidelines) prior to subject enrollment.

### Determination of CoMiSS

CoMiSS determination was completed as summarized in Table 1, with assessments involving crying, regurgitation, change in stool consistency, skin findings and respiratory symptoms. Bristol grading was used to evaluate the stool consistency [4].Type 1: separate hard lumps; type 2: sausage-shaped, but lumpy; type 3: like a sausage but with cracks on its surface; type 4: like a sausage or snake, smooth and soft; type 5: soft blobs with clear cut edges; type 6: fluffy pieces with ragged edges, a mushy stool; type 7: watery, no solid pieces, entirely liquid. Respiratory symptoms were defined as: (1) Mild: runny noses, sneezing; (2) Moderate: chronic coughing, wheezing; (3) Severe: wheezing or stridor, breathing difficulties. The GI physicians completed the scoring and complied information regarding their demographics including age, sex, birth history, and feeding patterns.

**Table 1:**
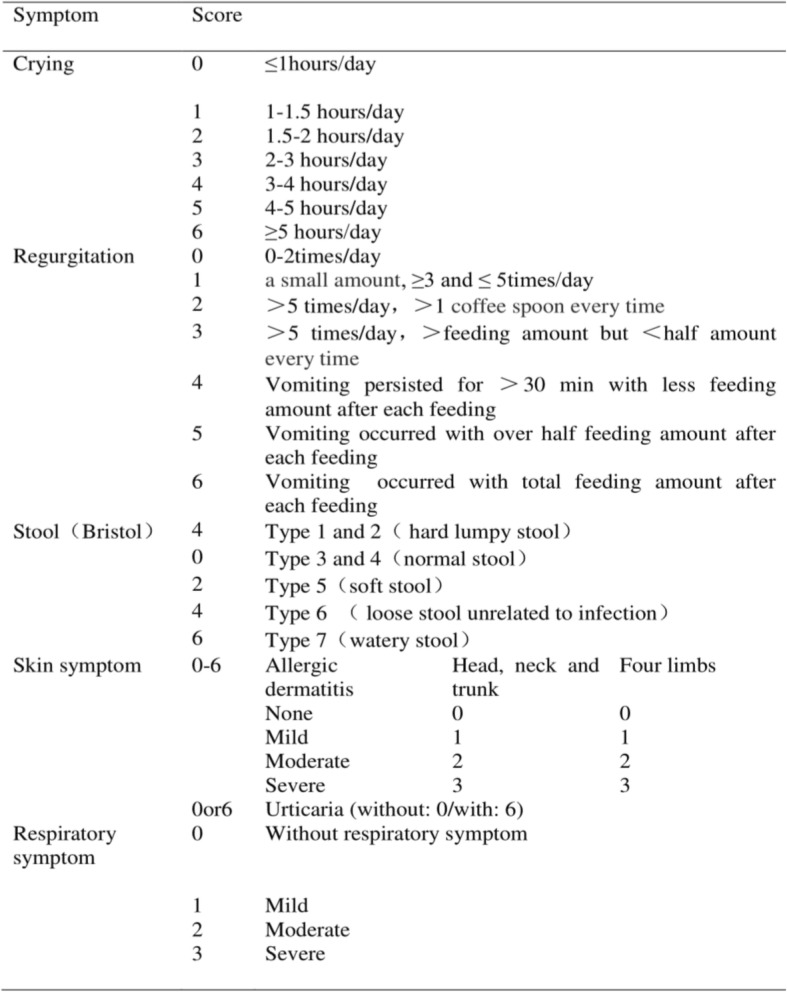
Symptom Score

### Dietary elimination

For those with suspected CMPA, a cow’s milk protein challenge test was performed. The breast-fed infants continued breast-feeding while their mothers avoided all milk and milk products from their own diet. In some breast-fed infants, proteins other than CMP (egg, soy, seafood) were avoided as well due to potential for allergic reactions [4]. Free amino acid-based formula was selected for the infants who had been fed with formula. After milk protein avoidance for 4 weeks, for the infants whose symptoms were improved or resolved, anti-allergic drugs (eg. chlorpheniramine) were discontinued, and the hormone and asthma drugs (eg. methylprednisolone, prednisone) were administered systemically for at least 1 week. The infants then returned to hospital to undergo OFC test. OFC test was not performed in the following cases: cow’s milk protein specific IgE (sIgE) ≥ 95%, positive predictive value (5.0 kU/L) [5], acute and chronic diseases, severe rash, moderate-severe malnutrition, congenital diseases and genetic metabolic diseases.

### Oral food challenge

Oral food challenge was performed according to the 2012 ESPGHAN GI Committee Practical Guidelines [6]. The infants were admitted to hospital and the intravenous access were established. The medical history were collected and physical examinations were performed for the infants. The parents of infants were fully informed of possible conditions and risks during OFC test and signed the informed consents. Adrenaline (1:1000), devices for electrocardiogram (ECG) and blood oxygen saturation monitoring and devices for cardiopulmonary resuscitation were prepared. A lactose-free CMP-containing formula was selected for OFC test. OFC test started at two hours after the last feeding of the infant. One drop of the formula was dropped onto the lips of the infant to observe for any reactions. If there was no reaction 15 min later, the feeding amount of formula was increased every 20 min, starting from 1 ml, followed by 3 ml, 10 ml, 30 ml and 100 ml (maximum feeding amount for a meal) (5 times in total). The vital signs and symptoms in skin, digestive and respiratory tract were observed, and were continuously observed for 2 h after the infant was given a maximum feeding amount. As soon as relevant clinical symptoms appeared, the test was immediately stopped and the symptomatic treatment was carried out accordingly. The infants who did not have an allergic reaction were discharged with close monitoring by parents at home and a plan to continue to consume at least 250 ml of lactose-free CMP-containing formula daily for the subsequent 2 weeks. If the infant receives any complementary feedings, these must be free of CMP. Other new foods or foods that might cause allergies were not added. The parents recorded the diet diary and reported any delayed reaction to the primary GI. The primary GI physician, who would make a clinical assessment.

### Assessment of results

If initial symptoms such as rash, vomiting, diarrhea, bloody stools, crying and breast refusal appeared again in the infected infants within 2 weeks of observation period after OFC test, it was determined that OFC test was positive, and CMPA was confirmed; if the above symptoms did not appear within 2 weeks, it was determined that OFC test was negative, and CMPA was ruled out and thus the infants were fed continuously with CMP-containing formula.

### Statistical analysis

SPSS19.0 software was used for statistical analysis of data. The measurement data were expressed as mean ± standard deviation($\overline{x}$ ±*s*)and the data were analyzed by independent-samples T test and chi-squre test. A *P*-value< 0.05 was considered significantly different. The ROC curve (Receiver Operating Characteristic curve) analysis was performed at the same time, and the best cut-off point defined by the maximum of Youden’s index was used to calculate the sensitivity and specificity.

## Results

### Population

**Fig. 1:**
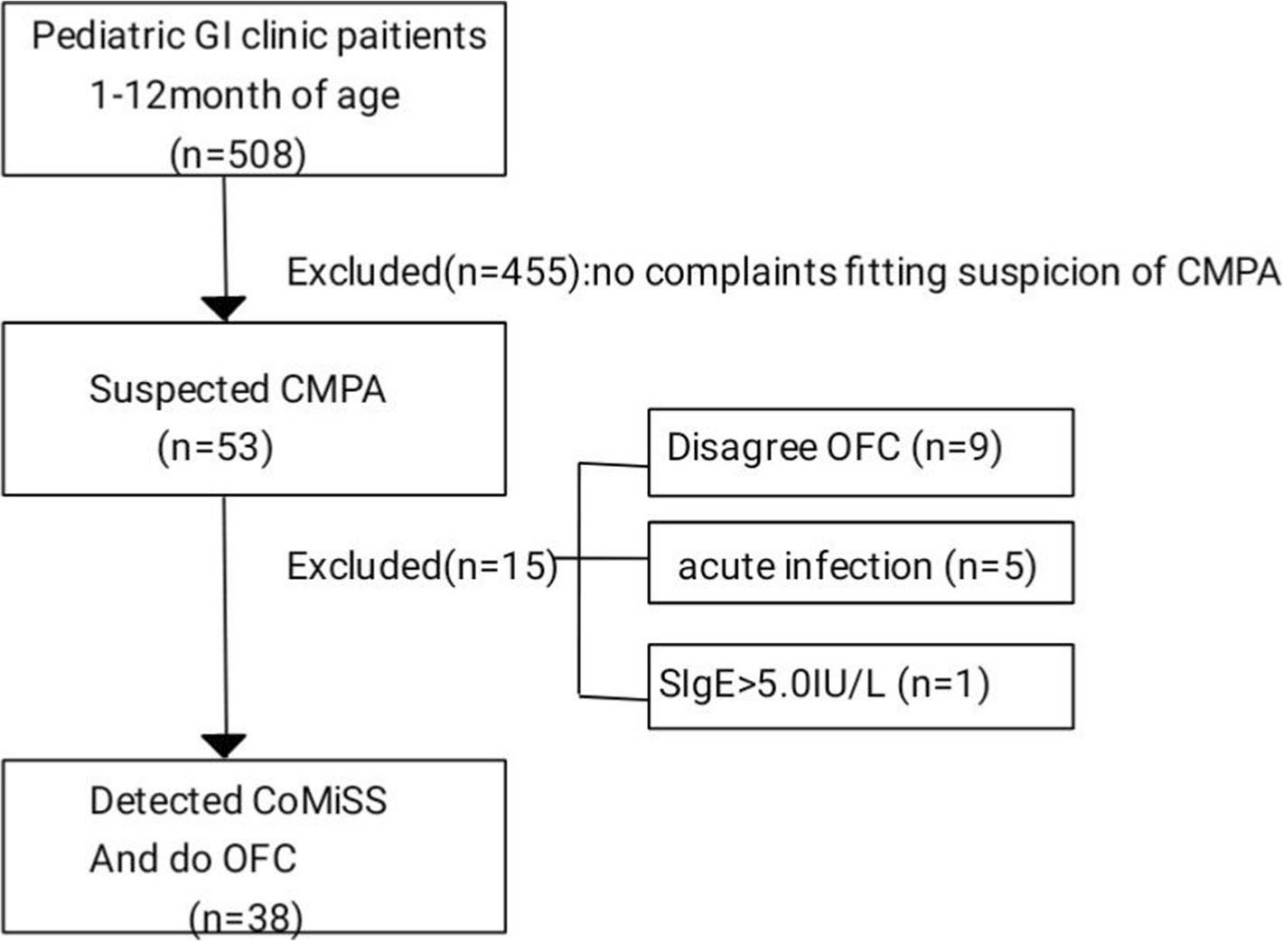
Flow chart of inclusion infants. Legend. GI = gastrointestinal. n = number of infants. CMPA = cow’s milk allergy. OFC = oral food challenge. CoMiSS=Cow’s Milk-related Symptoms Scores

As shown in Fig. 1, a total of 508 infants were enrolled. 53 infants(10.4%) were identified with suspicion of CMPA. After evaluation,15 infants were excluded, of which 9 caregivers refused to participate, 5 infants had acute infection, 1 infant’s cow’s milk protein specific IgE was higher than 5.0kU/L. Thus, a total of 38 infants were enclosed for CoMiSS and OFC.

### Demographics

We divided the suspected CMPA infants into two groups according to the results of OFC. Confirmed CMPA whose OFC were positive and No CMPA whose OFC were negative. As shown in Table 2, of 38 suspected CMPA infants, 24 had positive OFC while 14 had negative OFC. There was no significant difference in gender, age, gestational age at birth or feeding mode between the two groups.

**Table 2:**
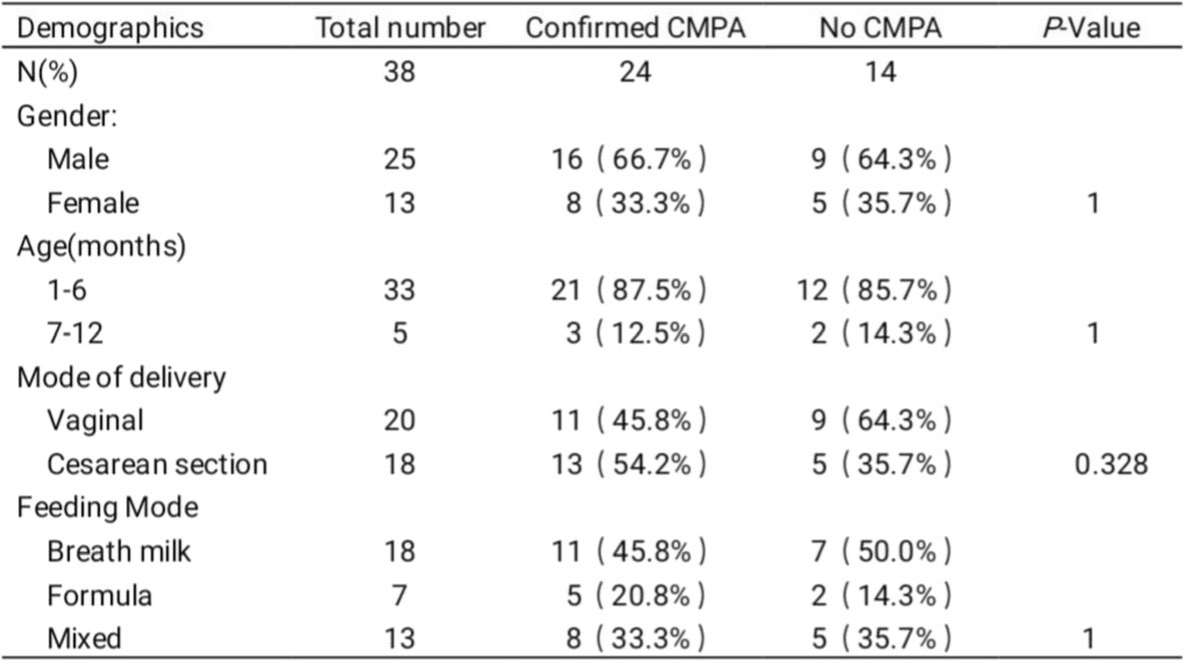
Clinical Characteristics of Infants with Confirmed CMPA (positive OFC) and those with No CMPA (negative OFC)

### Clinical manifestations

Among 24 affected infants definitely diagnosed with CMPA, there were 18 infants (75%) with eczema, 15 infants (62.5%) with bloody stools, 15 infants (62.5%) with diarrhea, 5 infants (20.8%) with regurgitation, and 3 infants (12.5%) with slow weight gain, 2 infants (8.3%) with repeated cough and asthma and 1 infant (4.2%) with crying. There was immediate reaction in 4 infants with rashes and delayed reaction in 20 infants during OFC in Confirmed CMPA group.

### Determination of sIgE

All 38 infants were tested for sIgE. In the Confirmed CMPA group, 10 of the infants (41.7%) had positive sIgE between 0 and 2.2kU/L while 14 infants (58.3%) had negative sIgE. In the No CMPA group, 3 infants (21.4%) had positive sIgE while 11 infants (78.6%) had negative sIgE.

### Determination of CoMiSS

**Fig. 2:**
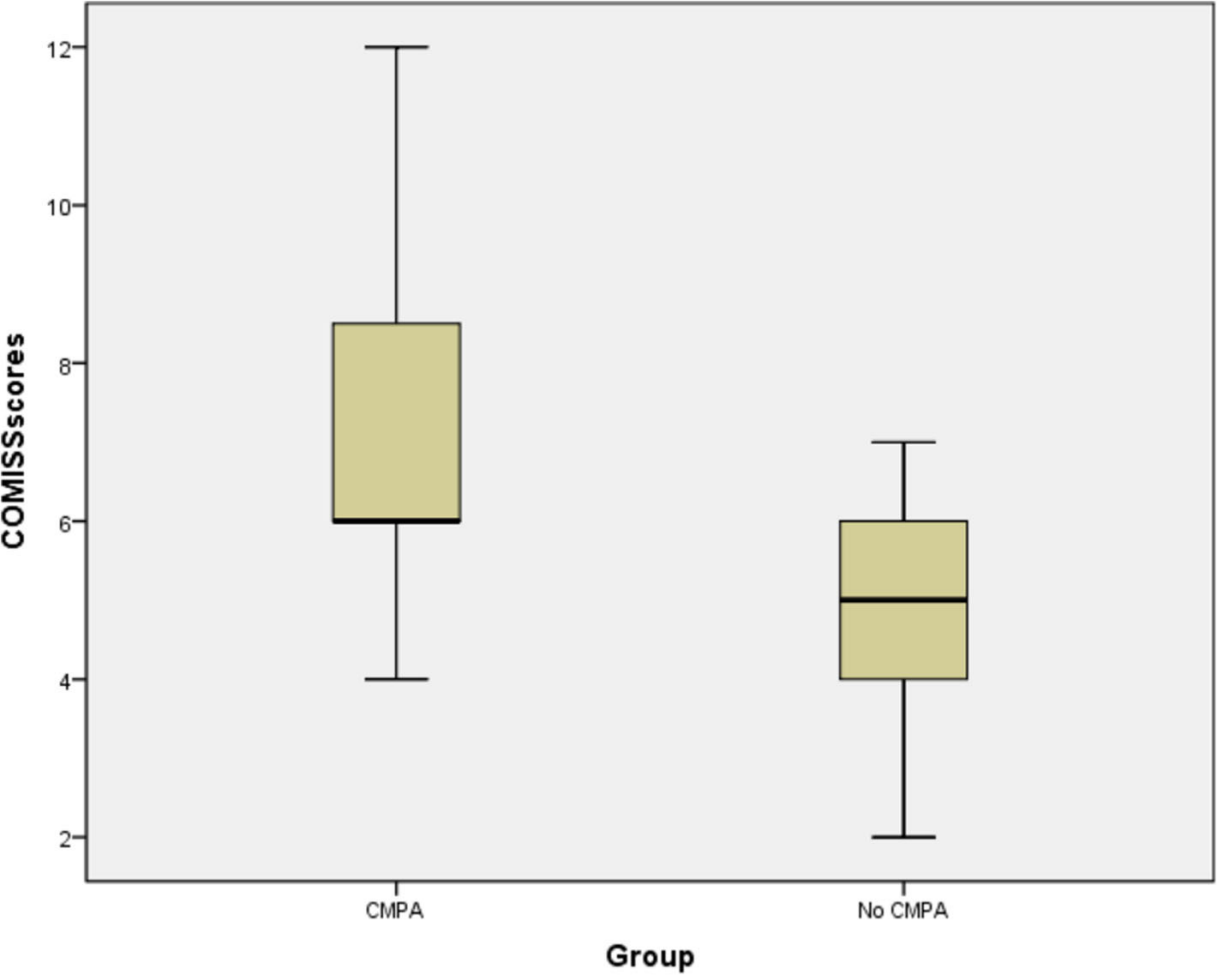
comparison of CoMiSS between two groups

As shown in Fig. 2, among 38 affected infants undergoing OFC testing, the average CoMiSS of the 24 infants in Confirmed CMPA group was 7.4 ± 2.3, while the average CoMiSS of 14 infants in No CMPA group was 4.1 ± 1.6. The results of rank sum test showed that there was a significant difference in CoMiSS between two groups (*F =* 2.13, *P* < 0.05).

### Analysis of sensitivity and specificity of CoMiSS

**Fig. 3:**
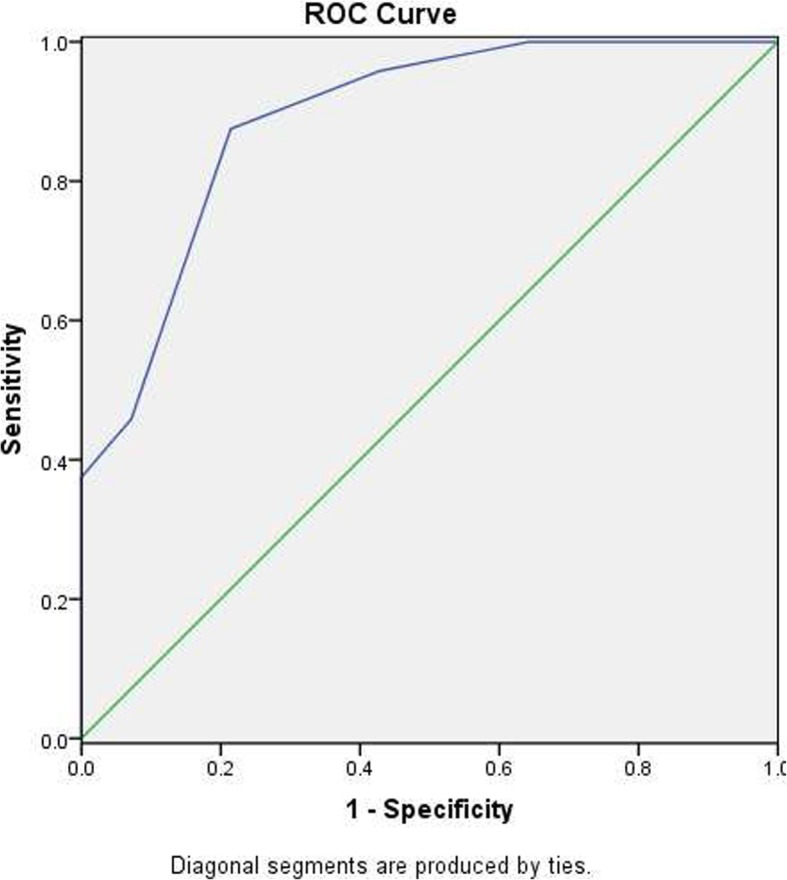
ROC curve analysis of CoMiSS

The ROC curve assesses true positive (sensitivity) versus false positive rate(1-specificity) for different thresholds of the CoMiSS. The area under the curve (AUC) of ROC curve was calculated and revealed that the best diagnostic cut-off point is 5.5. At this point, the AUC was 0.89, which corresponds to a 1-point ROC curve with 87.5% sensitivity and 78.6% specificity (95% CI: 0.722, 0.978). (Fig. 3).

### A retrospective power analyses

Considering the small number of patients for this study, we did a retrospective power analyses. If the best diagnostic cut-off point was 6, the coincidence rate was 84.2%.

## Discussion

CMPA is the most common food allergy in the infants, and the prevalence of CMPA in infants at the first year after birth is about 2 to 3% [7]. Its clinical manifestations lack specificity and easily leads to misdiagnosis. The tests include sIgE test, skin prick test, patch test and double-blind, placebo-controlled food challenge test. A recent meta-analysis showed [8] that the patch test has a sensitivity of 53% and a specificity of 88%, the skin prick test has a sensitivity of 88% and a specificity of 68%, and sIgE test has a sensitivity of 87% and a specificity of 48%. Another meta-analysis showed [9] that the self-reported prevalence of CMPA was 6% (5.7 to 6.4%), while the prevalence of CMPA confirmed by OFC test was 0.6% (0.5 to 0.8%). Double-blind, placebo-controlled food challenge test is the“gold standard” for the diagnosis of CMPA. But it’s complicated for physician to do in clinic and unsuitable for early diagnosis early. At the 2014 Brussels Expert Consensus, CoMiSS was proposed for early identification of CMPA. The more severe the symptoms, the higher the score and CoMiSS ≥12 points requires heightening alertness to CMPA.

This study showed that CoMiSS in Confirmed CMPA group was significantly higher than that in No CMPA group The average CoMiSS in Confirmed CMPA group was 7.4 ± 2.3 points. The ROC curve in this study showed that the best diagnostic cut-off point was 5.5, which was lower than CoMiSS≥12 points in the standard recommended by consensus. Of 24 confirmed affected infants, 15 (62.5%) had bloody stools. CoMiSS does not assign scores to bloody stool symptoms, which may be one of the major causes for low CoMiSS score in this type of affected infants. It is common for CMPA infants to manifest with bloody stools in clinic. This is the reason why our cut-off value is lower than that of the consensus.

As Vandenplas [3] said, CoMiSS is a simple score that will help clinicians to efficiently identify CMPA early, though it cannot be used as a diagnostic tool or a substitute for OFC test. Based on our data, we believe the use of CoMiSS ≥12 as a criterion for early identification of CMPA in Chinese infants may increase the risk of under-diagnosis. But on the other hand, if the criterion was adjusted to CoMiSS ≥6 according to our cut-off point, many infants with colic and reflux may reach the criteria and be falsely positive, thus decreasing the specificity of CoMiSS. In presumed healthy infants, the overall median and mean CoMiSS scores were, respectively, 3.0 and 3.7. The CoMiSS was greater in the Polish infants [10]. In our study, we didn’t determine the CoMiSS of presumed healthy infants in Chinese. It will be better to evaluate the CoMiSS and cut-off point if we combine the scores of health Chinese infants. Insufficient sample size and geographical constraints may shift the results. On the other hand, positive OFC is mainly based on the change of subjective and objective symptoms. The primary GI physician supervising the OFC is not absolutely blinded to the CoMiSS scores. It is easily to make the results to have bias. The application value of CoMiSS needs to be further confirmed by multi-center large sample studies [3, 11].

## Conclusions

CoMiSS is a simple and operable method to screen for CMPA, though there may be risk of under-diagnosis when CoMiSS≥12 is used as the criterion for early identification of CMPA in Chinese infants. More multi-center studies are needed to evaluate whether the factors such as bloody stool should be included in CoMiSS or CoMiSS≥6 can be used as the criterion for early identification of CMPA in Chinese infants.

## Data Availability

The data supporting the current findings are not publicly available since the database is currently not anonymous and contains all the patients’ names. However, it will be available upon request.

## Abbreviations

AUC: Area under the curve
CMPA: Cow’s milk protein allergy
CoMiSS: Cow’s milk-related symptom scores
OFC: Oral food challenge
ROC Curve: Receiver Operating Characteristic curve
sIgE: specific IgE
